# Vitality as a Germicide

**Published:** 1891-10

**Authors:** Thomas Fillebrown

**Affiliations:** Boston


### VITALITY AS A GERMICIDE.[1]

BY THOMAS FILLEBROWN, M.D., D.M.D., BOSTON.

THE reign of antisepticism has long been autocratic. Its disciples and advocates are enthusiastic and opinionated, consequently they are impatient of any expressions of possible doubt of the entire correctness of their positions. Hence it requires some consid-

[1] Read before the American Academy of Dental Science, Boston, June 3, 1891.

erable temerity to publicly argue against antisepticism, and put forward asepticism as the more important principle upon which surgical success depends, yet I feel that it is quite time to call the attention of the dental profession to the probability of the doubt, and ask a consideration of the latter position. More than fifteen years ago, at a meeting of the Merrimac Dental Society, I expressed the opinion that the power of micrococci over decay of the teeth was over-estimated; that bacteria alone in contact with the teeth were not sufficient to cause decay; and that certain favorable conditions must be present to enable them to act and cause destruction. I stated my belief that the essential condition was weakened structure, arising either from imperfect formation or from degeneration from some secondary cause; that until the vitality of the structure was weakened the bacteria were powerless. My observations during succeeding years, together with the results of the investigations of bacteriologists and pathologists, have strengthened my convictions, and to-day I feel that I am one of a "powerful remnant," to quote Matthew Arnold, which is sure to become a ruling majority.

Of general surgery I do not presume to have sufficient practical knowledge to enable me to speak with authority, and shall let surgeons speak for themselves.

For facts regarding bacteria I shall rely upon the investigations of experts in that branch.

Of results in treatment of the teeth, and the larger field of oral surgery, I feel that my observations are of some value. It has been proved beyond a doubt that bacteria cause fermentation, putrefaction, and many diseases. It has also been proved and is universally admitted that fermentation and putrefaction never occur except by the action of bacteria; also that certain chemicals and extremes of heat and cold will destroy both the plants and their germs. But it is not generally understood or admitted that chemicals of sufficient strength to destroy the bacteria and their germs will seriously injure or destroy the tissue with which they may come in contact, nor has vitality of the tissues as a practically effective force to destroy the bacteria been sufficiently recognized. Hence vitality as a germicide is what I propose to consider at this time.

The work of the discovery and the identifying and classifying of microbes has been great, and has occupied a long time.

In 1516, Magnatus claimed that the air was full of infectious miasms; Wiseman, in 1692, held the same view, and in 1706, Parmanus speaks of a lotion which, applied to wounds, resists putrefaction and takes away the pain and inflammation from the wound. That yeast consisted of microscopic cells was proved in 1680 by Leeuwenhoek. That these cells were plants was shown by Tour in 1836. In 1837, Schwann found organic germs in the air, and connected them with the processes of fermentation and putrefaction. It still remained for Pasteur, in 1857, to first observe and explain the mechanism of the relations between fermentation and the vital processes of micro-organisms. Since that time knowledge of bacteria and their action has been rapidly acquired and classified, and now bacteriology is a well-established branch of science.

Delpech, Dupuytren, and Offenbach developed the "subcutaneous method" of operating, which proved so successful. This led to the efforts of J. Guérin and Chassaignac to convert open into subcutaneous wounds.

Soon after Pasteur's researches of 1857, it came to be accepted as a fact that all inflammations as well as fermentations and putrefactions were the result of the action of bacteria; hence, from these premises, Lister's theory that the exclusion of germs from a wound would prevent inflammation and degeneration, and allow immediate healing, was logical. As a result we have, in 1872, Lister's antiseptic treatment of wounds, which has become so famous and productive of so much good. The benefit of Listerism on the progress of surgery cannot be over-estimated, but it should be borne in mind that it was developed at the same time that greatly improved methods of operating were being introduced and the importance of greater cleanliness was being recognized. Lister's genius led him to combine with his great ability the powerful adjuvants of antisepticism and cleanliness, but his modesty led him to ignore to a great degree his consummate skill as a factor in his system. Cleanliness, as such, appeared as only a necessary incident, while antisepticism was to him the basis and framework of his superstructure.

For fifteen years the proposition that antisepticism was the one essential principle on which surgical success depended was hardly questioned. Antiseptic surgery has in theory, though not in practice, ignored to a great degree the importance of three points which, with drainage, have together been the real foundation of the success of modern surgery,—viz., skill, cleanliness, and that other factor so long called "condition of the patient;" this, now, in antiseptic nomenclature, is included in "proper selection of cases," "early operations," etc. All these are circumstances which affect the one condition necessary to success,—vitality.

The bad case makes too great a demand on the vital strength of the patient, and he dies.

The early operation has the full vital power to resist the shock and make the repair.

Listerism, as a system, is based upon the antisepticism of chemical poisons, while the rational system is based upon the asepticism of cleanliness.

The principles upon which Listerism is based are, in their order, antisepticism, drainage, condition of the patient, skill.

The principles upon which the rational method rests are vitality, skill, asepticism, drainage.

Good nursing and nourishment are concomitants in either case.

A further observation of facts will make plain that antisepticism cannot be relied upon, and that it is the asepticism, which is the result of the antiseptic practice, which gives success.

The researches of scientists has established the fact of the existence of almost innumerable kinds of bacteria, varying in their degree of vitality, which cause the disorganization of organic substances, and also forms of pathogenic bacteria, which are the causes of infectious diseases.

The vitality of the disorganizing bacteria is much less than that of the healthy normal cell of animal tissue. The pathogenic germ requires some favorable conditions, not yet well understood, to give it power within the system.

The air around us, the dust under our feet, the food which we eat, and often the water we drink are filled with various kinds of these bacteria. Hence no living being enjoys immunity from their presence.

Although now almost universally abandoned, the use of the carbolic acid spray was at first deemed an essential and indispensable adjunct in Listerian surgery. The carbolic acid (five per cent.), diluted by the steam from the atomizer, and then further diluted by the air into which it was injected, became so reduced that the final strength, which comes into contact with the bacteria floating in the air, is at most only a fraction of one per cent.

The puerility of this spray as a germicide is shown by comparison with facts established by recent observations made in laboratories.

Koch found that the bacteria in broken-down beef-tea were not destroyed in two hours in a four-per-cent. solution of carbolic acid. De la Croix found the same alive after immersion in a ten-per-cent. solution. Koch found that a one-per-cent. solution did not affect the anthrax spores in fifteen days; a two-per-cent. solution did not destroy in seven days. Hence a less than one-per-cent. spray for two hours would be harmless to the spores.

Dr. Prudden, of the New York College of Physicians and Surgeons, demonstrated—

1. That freezing killed a large number of bacteria.

2. That if the vitality of the bacteria is reduced by exhaustion of nutriment in the culture, great numbers are killed by freezing, but if they are vigorous this will not be the case.

3. Some species are capable of growth after long exposure. The typhoid bacillus resists freezing temperature for a long time.

4. In any given culture the resistance of individual bacteria varies greatly.

5. Alternate freezing and thawing destroy more effectually than constant cold.

The Société Médico-Chirurgicale, of Lyons, conducted a long series of experiments which established the following conclusions:

1. The action of steam under pressure is absolutely efficacious between 112° and 115° C. It destroys the most resistant germs after an application lasting fifteen minutes.

2. Hot air and superheated steam are of less value. At 130° C. certain germs escaped when the application of heat was prolonged twenty minutes!

Dr. Ernst, of the Harvard Medical School, in a paper read before the Harvard Odontological Society, showed that the only sure way of sterilizing was by the application of steam at 212° F. for two hours, as applied in Arnold's sterilizer, and suggested it as an available means of applying the steam for that purpose.

Now, if these observations are of any value and the conclusions reached are facts, how utterly useless it is to think for a moment of producing anything like antiseptic conditions in operations, and how evident it is that surgical success depends upon some other more important condition!

Antiseptic theories were promulgated and the system established when bacteria were considered to be the immediate cause of sepsis in all of its forms. Later discoveries have shown that bacteria have no power over living tissue, but that among the products of putrefaction are alkaloids called ptomaines, which are violent poisons, and which are readily taken into the circulation when brought into contact with an open wound, and cause blood-poisoning effects. A minute amount of the alkaloid will give rise to serious consequences. This is unquestionably the source of poisoning from eating canned goods, either meats or vegetables, or ice-cream. The vitality of ptomaines resists a high degree of heat, much greater than is necessary to destroy all forms of bacterial life; hence the matter is made very plain. The meat has, before canning, been exposed to infection and ptomaines developed. In canning, the meat is subjected to a heat quite sufficient to kill the microbes but leave the ptomaines undestroyed ready for work, and colic, vomiting, diarrhœa, lassitude, emaciation, and all the long line of ills which follow blood-poisoning are the result.

The action of these ptomaines, independent of any germs, may be learned from the following extract from Senn's "Surgical Bacteriology:"

"That putrid substances injected directly into the circulation produce symptoms of septic intoxication has been known for a long time, and the extensive researches of Panum threw additional light on the subject. It was believed that putrid materials, when introduced into the organism, induced a process of fermentation, to which were attributed the most constant post-mortem appearances found in septicæmic subjects,—fluidity of the blood and softening of the tissues. That these changes were not necessarily caused by the action of living micro-organisms was determined by experiments; as the introduction of putrid blood, or meat infusion that had been boiled for a considerable length of time, produced toxic symptoms, and, when a sufficient quantity was used, death and identical pathological changes in the blood and tissues, as in cases of true sepsis.

"Semmer (*Virchow's Arch.*, Bd. 83) gives an account of the action of septic substances as studied experimentally by Guttman in the pathological department of the veterinary school at Dorpat. The experiments were made with putrid substances, products of inflammation, septic blood, and cultivations of septic bacteria. These researches showed that a chemical putrid poison is formed in putrefying substances, and that a certain quantity of such poison produces symptoms of sepsis and death in animals. The blood of animals killed with such putrid poison was found to possess no infective qualities, and the usual putrefactive bacteria are destroyed in the blood, and only appear again after the death of the animal.

"Buiger and Maas have rendered valuable service in the chemical isolation of ptomaines from putrid substances, and the results of their inoculation experiments established more firmly the fact of putrid intoxication by ptomaines. The number of bacteria in rabbits killed by septic infection is so great that death may ensue from mechanical causes, while in fatal cases of sepsis in man the number is often so small that it seems natural to suppose that the micro-organisms are capable of producing some poisonous substances which destroy the patient before they have time to multiply to the extent observed in the septicæmia of rabbits and mice."

The statement has been made in the New York Odontological Society that the blood and tissues are full of bacteria. If this were the case, and it were true that bacteria caused putrefactions, we all should be indeed not living, but dead and putrefying.

The absurdity of the statement is shown by the following experiments:

Hauser (Vorkomen von Micro-organismen in "Lebenden Gewebe gesunder Thiere," *Archiv für Experimentelle Pathologie and Pharmacologie*, Bd. xx. pp. 162–202) has made a number of carefully-conducted experiments to show that no microbes exist in healthy animals.

The experiments consisted principally in procuring tissues prone to fermentation, as parts of internal organs, blood, etc., and protecting them against infection from without. He kept the specimens in rarefied air, in filtered air, hydrogen, oxygen, carbonic acid gas, and water, and in various artificial culture-soils, at a temperature favorable to putrefaction, but in all instances in which the specimens remained uncontaminated no putrefactive changes were observed.

By this method he believed he was able to demonstrate that tissues taken from healthy animals immediately after death contained no bacteria, since it is well known that if the specimen were not perfectly sterile putrefaction would have taken place. The author did not only appear to demonstrate that living tissues contained no micro-organisms, but he also ascertained that the preserved sterile organs in time underwent a sort of regressive metamorphosis similar to that which takes place in the absence of micro-organisms, and what is of especial interest, that the product of such processes of resolution possess no poisonous properties whatever.

Watson Cheyne (on "Suppuration and Septic Diseases," *British Medical Journal*, March 3, 1888) found, in his experiments on the presence or absence of micro-organisms in the living tissues, that, while germs were absent when the animal was in a good state of health, yet if the vitality of the animal was depressed, say by administering large doses of phosphorus for some time, organisms could be found at times in the blood and tissue of the body.

Nelson, in a paper read before the American Academy of Medicine, and approved for publication, on "Micro-organisms and their Relation to Disease," says,—

"It has been a widely-disputed question as to whether bacteria ever occur in the animal in a perfectly healthy state; the affirmative view having been taken by Billroth and some others; but it is denied by Koch, by Pasteur, and by Ehrlich, who state that they have never detected bacteria in the healthy animal. The failure of putrefactive bacteria, according to experiments, would go to show inability to struggle against the normal cells indigenous to the soil upon which they were planted. Some bacteria showed power of existence only in tissue in which vitality had entirely ceased, while others seemed to possess the power of existence in the presence of the animal cells when the latter suffered from impairment of nutrition, and the tide of life was turning against them. Abnormal composition of the blood seemed to favor the development of some bacteria, after they had found their way into the tissues."

The relation of pathogenic germs to the healthy tissues is shown by the following, from Senn's "Surgical Bacteriology" (p. 25, *et seq.*):

"From these remarks it is reasonable to assume that pathogenic germs may exist in the healthy body without necessarily giving rise to disease, especially if, as is well known, they are being constantly eliminated through the excretory organs."

Fodor ("Bacterien in Blute Lebenden Thiere," *Archiv für Hygiene*, Bd. ix. p. 129) introduced directly into the circulation of rabbits pathogenic bacteria in order to study their effects on the tissues and manner of elimination. As a rule, he found that they had completely disappeared from the blood after twenty-four hours. No culture-experiments were made less than four hours after inoculation.

He believes that the microbes are removed by leucocytes. He affirms that, as a rule, pathogenic germs are not present in the healthy organism, as he found the blood of healthy rabbits, without exception, sterile; and only in exceptional cases was he able to demonstrate the presence of bacteria in animals killed, even where the examination was postponed until after putrefaction had set in.

The phenomenon, familiar to every one, of blood effused into a contused wound being absorbed and the parts recovering perfect health, without any signs of putrefactive changes, is a clear proof that bacteria are not in healthy blood. Another proof is the fact that cysts form and contain tissue which is taken out of the circulation and has even undergone suppurative changes, and exist long as cold abscesses without a sign of putrefaction or even inflammatory action, and most of such cases will heal kindly if opened, drained, and kept clean.

The results of modern abdominal surgery show that cleanliness, and not antisepsis, is the right bower of success.

I believe the best record that Listerism has shown is to reduce the mortality to about eight per cent., while Lawson Tait, in his last thousand reported cases, without a pretence of antisepticism, but an entire avoidance of it, reduced the mortality to about three and one-half per cent. Mr. Tait had a run of one hundred and thirty-nine cases without a death, while Mr. Thornton, the most persistent apostle of Listerism of the present time, had only forty-eight cases without a death. The testimony of so successful an operator as Mr. Tait upon the harmlessness of germs upon the living tissue is worthy of attention and consideration.

I will quote in full his language upon this subject:

"For my present purpose, therefore, it is enough for me to assume, as I do most fully, that the germ-theory has been completely substantiated, and that no known putrefaction does occur, save by the admission of resting spores, or swarm spores of some of the many minute living organisms which are invariably associated with putrefactive changes.

"But concerning this, there is another constant position associated with this phenomena. The materials upon which the experiments have been made, of infinite variety of kind and constitution, have all been dead, and no one has yet pretended that, by the admission of germs to living matter, he has produced the phenomena of the putrefactive changes which constantly result in matter which is dead. To quote the apt illustration given by Dr. William Roberts in his masterly exposition of this most difficult subject, the ordinary hypodermic morphia-syringe will inoculate inevitably a sterilized solution of dead organic matter, but among the hundreds and thousands of hypodermic injections which are made daily, no one has yet declared a single instance of putrefactive changes resulting from it in the healthy or even in the diseased human body.

"It will, therefore, be seen that the application of the facts of the germ-theory of putrefaction to the phenomena of disease of living tissue is met at once by an overwhelming difficulty, to the removal of which none of the adapters, so far as I have seen, have as yet applied themselves.

"Granting that the same germs which would inevitably produce putrefaction in a dead infusion of beef are constantly admitted to wounds, there is not the slightest particle of evidence that they do produce any change whatever upon living tissue, still less is there any evidence that the changes which occur in the numerous varieties of what we call blood-poisonings, even when they are of an undoubtedly local origin, have the slightest analogy to those seen in a putrefying dead infusion.

"The mere presence of bacteria in the fluids of wounds, or in fluids enclosed in cavities, while offering many difficulties to the adapters of the germ-theory, prove nothing for their positions until they have shown that these organisms ever do occur in fluids or tissues which are truly living.

"The difficulty, therefore, is this: that what we call vital action, for want of a name based upon a better understanding of what it is, places living tissue in an altogether different category from tissue in which the phenomena of life are no longer present.

"Now, this is consonant with every-day experience. If a decaying hyacinth-bulb or a rotten apple be examined, the presence of the minute forms of life is found to be absolutely confined to those parts where the changes have been effected, while those parts to which the rot has not extended are found absolutely free from them, and the difficulty of the adoption of the germ-theory is simply this: that its advocates have assumed that the invasion of the germs is the cause of the decadence of the vital phenomena and the ultimate death, while there is the alternative,—still undiscussed, and certainly undismissed,—that the decadence of the vital powers, due to some cause possibly yet unknown, is that which gives the germs their potential ascendency, and enables them to do what, during full vital action, they are wholly unable to effect.

"If the view of the germ-theorists were correct, we ought to expect that no operation could be done successfully without rigid antiseptic precautions. The slightest cut of the skin ought to be followed by septic poisoning. There ought to be no difference in the mortality of operations in small and large hospitals, in town and in country. In fact, if germs could have had the unbounded influence which is claimed for them by many antisepticists, surgery must long ago have been an extinct art, if, indeed, it could ever have struggled into existence.

"The uniform experience of operating surgeons has taught them that the success of their work will depend upon three factors,—the condition of the patient, the condition of his surroundings, and the nature and extent of the operation performed.

"Of these three, undoubtedly, the most uncertain factor is the first. What condition of the system it is which is favorable to operations is almost unknown. I must base my conclusions chiefly upon my own work, and in my special operation of ovariotomy I am perfectly certain that apparent perfect health is by no means a certain indication of a power of resistance to those conditions, whatever they be, which result in so-called septic poisoning.

"The second of these factors, the condition of the surroundings of the patient, contains elements of far greater certainty. It has approached the position of a statistical law that the death-rate is in constant harmony with the density of the population, and when the density exceeds a certain minimum of safety there are introduced specific septic diseases, as typhus fever, which are wholly unknown under other conditions, and which, even after the danger density has been reached, attack certain individuals only, and not all, for reasons which can be expressed only by saying, as I have already said, that the living tissues of those affected could not and did not resist the septic influence.

"Every advance we make in sanitation shows that this factor, the condition of the surroundings of the patient, is of extreme importance.

"The third factor which influences surgical success is the extent and importance of the operation performed. Everybody knows that while amputation of a finger is probably fatal in not more than one in ten thousand cases, nearly one-half of all amputations of the thigh die. Now, if the adaptation of the germ-theory to surgical practice were as promising and legitimate as some of its supporters allege, we should have had the remarkable result, previous to its application, that amputation of the finger and the thigh ought to have approached one another in mortality to an infinitely larger extent than they have done.

"If the contact of a bacterium germ upon a wound could be the source of blood-poisoning, then the size of the wound and the nature of the operation could make but small difference in the result, and a wound into the theca of a finger tendon and one of a similar size into the peritoneum of another patient in the same ward ought to have very similar risks. But, as a matter of fact, they do not, and we are forced to the conclusion that, even if bacteria germs lighting on the wounds are the cause of much surgical mortality, the power of vital resistance by the tissues, or the condition of the patient and the nature and extent of the operation are of infinitely greater importance as factors in the general result."

No words I can add can make it clearer that vitality—life—is the one effective germicide upon which all surgical success depends. There are, so to speak, two kinds of vitality: the first, vitality of construction; the second, vitality of resistance.

The first is marked by a quick, full pulse, arteries and veins over-full of blood, a well rounded form that counts well in avoirdupois; this succumbs easily to destructive influences. The vitality of resistance holds on to life under deprivations, hardship, exposure, and famine. This is the vitality that those possess who live on through destruction and pestilence when thousands around them die.

In 1827, Bichat wrote, "Life is the sum of the functions which resist death." This life, it appears to me, consists of two forces: first, a power of forming, and second, a power of resisting. The first exerts the positive force of building up during the period of growth and keeping up the repair during middle-life and declining years. The second is a guard ever watchful to keep the system in order, and to stand always ready to resist the ingress of disease in all its forms. This resisting vitality is the true germicide. In the presence of this vitality no bacterium or germ can thrive or live.

Oral and dental surgery furnish illustrations of the positions taken in this paper. Wounds of any character of the mouth rarely suppurate, and but a very small proportion even inflame. Of the vast multitude of teeth that are extracted every year, not one case in ten thousand is followed by septic degeneration. And where such degeneration does follow there is evidently impaired vitality. If bacteria in wounds were alone sufficient to produce sepsis, what dire results must follow so frequent operations as extractions! Complete drainage and constant washing with the secretions of the mouth keep the wounds aseptic, and hence the favorable results.

Again, the treatment of pulp-canals is an illustration. If a pulp is *completely* removed by operation, the cavity dried, and immediately filled, trouble will never occur in a healthy subject. If a canal is already septic and even causing irritation about the root of the tooth, there is no surer way of effecting a cure than to open the cavity, thus giving drainage, and allowing the scavengers to consume the *débris*.

Before the science of antiseptics was so fully developed, this expectant treatment was taught and successfully practised. Pulpcanals being closed cavities in hard tissue, impermeable to fluids, the use of strong antiseptics was not only unobjectionable, but of decided benefit in hastening the cleansing process. And the asepsis of cleanliness brought the successful result.

Finally, I will quote Professor Miller, who closes an exhaustive discussion of the prophylactic and curative effect of antiseptics by saying, "Under all conditions, however, the chief thing is the thorough mechanical cleansing of the teeth."

